# Left‐right asymmetry influenced the infarct volume and neurological dysfunction following focal middle cerebral artery occlusion in rats

**DOI:** 10.1002/brb3.1166

**Published:** 2018-11-19

**Authors:** Zhiyong Zhai, Juan Feng

**Affiliations:** ^1^ Department of Neurology Shengjing Hospital of China Medical University Shenyang China

**Keywords:** asymmetry, cerebral ischemia‐reperfusion, middle cerebral artery occlusion, rats

## Abstract

**Objective:**

To investigate the differential effects of left versus right cerebral hemisphere on the infarct volume and behavioral function following focal cerebral ischemia in rats.

**Methods and Materials:**

Middle cerebral artery occlusion (MCAO) was induced in the right‐handed rats by filament insertion for 1.5 hr, and then reperfusion was established according to Zea‐Longa method. A total of 36 male Sprague Dawley rats were randomly divided into a left MCAO group or a right MCAO group. The modified neurological severity scores (mNSS), tapered beam‐walking test, and Morris water maze experiment were all carried out to evaluate the sensorimotor and cognitive outcomes at the 1d, 3d, and 7d after MCAO, respectively. Infarct volume of the brains was measured by triphenyltetrazolium chloride (TTC) staining.

**Results:**

The sensorimotor function was more worse in the left MCAO group than that in the right MCAO group at the 1d, 3d, and 7d after MCAO (*p* < 0.05). While the cognitive function was much better in the left MCAO group than that in the right MCAO group at the 1d and 3d after MCAO (*p* < 0.05). But no significant difference was achieved in cognitive function between the two groups at 7d after MCAO (*p* > 0.05). There was no significant difference in total infarct volume between the two groups at the 1d, 3d, and 7d after MCAO, respectively (*p* > 0.05).

**Conclusion:**

The infarct volume is not affected significantly by the left or right MCAO model in the early days. The lesions in the left hemisphere produce more severe sensorimotor impairments, while more severe cognitive impairments are produced by the right hemispherical lesions. These findings suggest that it is structural and functional asymmetry between the two hemispheres other than infarct volume that affects the outcomes of rat MCAO**.**

## 1 INTRODUCTION

The middle cerebral artery occlusion (MCAO) in rats is commonly used to study pathophysiology and therapeutic approaches in cerebral ischemia because of its minimal invasion. MCAO can be introduced by inserting a monofilament into the cervical internal carotid artery (ICA) and advancing it intracranially to block blood flow into the middle cerebral artery (Longa, Weinstein, Carlson, & Cummins, 1989). Many reports have demonstrated that sensorimotor and cognitive functions are significantly impaired after MCAO. Improvement of sensorimotor and cognitive functions often occurs along with reduction of infarct size after neuroprotective treatments (Lee et al., 2014; Pétrault et al., 2017; Zhang et al., 2017). Most researches about the size and distribution of infarct by MCAO have focused on species of animals, sex, weight, different intraluminal filaments, the loci of occlusion, thread insert length, reperfusion periods, and so on (Lee et al., 2014; Schmid‐Elsaesser, Zausinger, Hungerhuber, Baethmann, & Reulen, 1998). And the side selection of the model depends mainly on personal habits.

Laterality of the brain (handedness, lateralization of speech, and other left to right differences), once believed to be a unique human characteristic, has been found to be widespread among vertebrates (Aizawa et al., 2005; Halpern, Güntürkün, Hopkins, & Rogers, 2005; Vallortigara, 2000). However, the differences between the left and right MCAO models in infarct volume and neurologic function are still not so clear. Gao and Zhang (2008) have previously reported the infarct volume in the dominant hemisphere was larger than that in the non‐dominant hemisphere in adult rats. However, there are evidences demonstrating that asymmetry of biochemical response and behavioral to cerebral infarction is not attributed to differences in the infarction in either hemisphere (Robinson, 1979; Robinson & Coyle, 1980).

In the present study, we tested infarct volume, neurologic function in either hemisphere and the possible associations between infarct volume and neurologic function at different time points.

## 2 METHODS

### 2.1 Animals

The experiment was conducted on right‐handed adult male Sprague Dawley rats (250–280 g). The paw preference was assessed according to a modified quadrupedal food‐reaching test (Tang & Verstynen, 2002). The rats were randomly separated into two groups: the left MCAO group and the right MCAO group. The animals in each group were then randomly assigned to the following groups: (a) 1d after MCAO (*n* = 6); (b) 3d after MCAO (*n* = 6); and (c) 7d after MCAO (*n* = 6).

All experiments involving rats were performed in accordance with protocols approved by the Institutional Animal Care and Use Committee of China Medical University (permit No. 2015PS326K) and the National Institutes of Health Guide.

### 2.2 Induction of focal cerebral ischemia‐reperfusion injury

Middle cerebral artery occlusion was induced to create a focal cerebral ischemia‐reperfusion injury in adult male rats as previously described (Longa et al., 1989). Briefly, the rats were anesthetized with 10% chloral hydrate intraperitoneally at a dose of 350 mg/kg. The common carotid artery was exposed, and a nylon suture (0.26 mm diameter) coated with polylysine was inserted into the ipsilateral internal carotid artery through the external carotid artery and advanced until it occluded the origin of the MCA. The completeness of occlusion and reperfusion were confirmed by laser‐Doppler flowmetry (LDF). For placement of the LDF probe, a hole was drilled into the parietal bone of the damaged hemisphere at anteroposterior −2.0 mm and mediolateral +6.0 mm from bregma. At least a 70% reduction in blood flow values in the middle cerebral artery area relative to baseline is needed to confirm occlusion by LDF. Then the intraluminal filament was carefully withdrawn to establish reperfusion after a 90 min of MCAO. Rats that did not demonstrate a rapid restoration of the LDF signal during reperfusion were excluded. The blood flow in the middle cerebral artery area was continuously measured from before the onset of ischemia until 30 min after reperfusion. Anesthesia was discontinued after 30 min of reperfusion. The body temperature was maintained at 37 ± 0.5°C by a thermoregulated heating pad throughout the surgery.

### 2.3 Behavioral tests

Rats were tested for neurological deficits at the 1d, 3d, and 7d after MCAO, respectively. The mNSS were performed to assess comprehensive sensorimotor function of rats as a previous study described (Hunter et al., 2000). The mNSS ranged from 0 to 18 (normal score, 0; maximal deficit score, 18), including tests of motor (muscle status, abnormal movement), sensory (visual, tactile and proprioceptive), reflex (pinna, corneal, and startle), and abnormal movements. A higher score represents more severe injury. The specific balance function was evaluated by tapered/ledged beam‐walking test (Zhao, Puurunen, Schallert, Sivenius, & Jolkkonen, 2005). The walking process was video‐recorded and analyzed by calculating the foot fault score according to the following formula: Foot fault score = number of slips/number of total steps.

Spatial learning ability was measured in a Morris water maze as previously described (Hosseini‐Sharifabad et al., 2011). Live video was recorded to evaluate the escape latency (time to locate the platform).

Each rat was scored by two investigators blindly among different experimental groups.

### 2.4 Infarct volume measurement

The rats were sacrificed after behavioral tests at the 1d, 3d, and 7d, respectively. The brains were excised, frozen at −20 degrees, and were cut into consecutive 2.0‐mm‐thick coronal sections. The sections were then stained with 2% TTC at 37°C for 30 min. Digital images were taken and all infarct measurements were performed by a blinded observer using a computerized image analyzer (ImageJ, NIH). The size of infarct regions was calculated by the following formula: *contralateral hemisphere volume−(ipsilateral hemisphere volume−measured infarct volume)* (Swanson, Shiraishi, Morton, & Sharp, 1990). The infarct volume was calculated using the corrected percentage of infarct volume: *(contralateral hemispheric volume−ipsilateral non‐infarcted volume)/contralateral hemispheric volume* (Matsuda, Sakakima, & Yoshida, 2011).

### 2.5 Statistical analysis

All data are presented as mean ± *SD*. Statistical differences between the left MCAO group and the right MCAO group were assessed using the Student's two‐tailed *t* test. One‐way ANOVA followed by Student‐Newman‐Keuls (SNK) post hoc analysis was used for multiple comparisons among different time points (1d, 3d, and 7d). A *p* value of <0.05 was considered statistically significant. SAS 9.1 software (RRID: SCR_008567) was used for statistical analysis.

## 3 RESULTS

A total of 45 rats were used. Five rats died after MCAO surgery, and four rats were excluded due to too low drop in Doppler signal. In the process of making the model, removed the model rats of death, and added new rats randomly to ensure the number of animals in each group.

### 3.1 Neurological deficits

All rats exhibited neurological deficits. The most severe neurological deficits were present at 3d after MCAO. However, all rats showed improvement in behavioral outcomes at 7d after MCAO.

**Figure 1:**
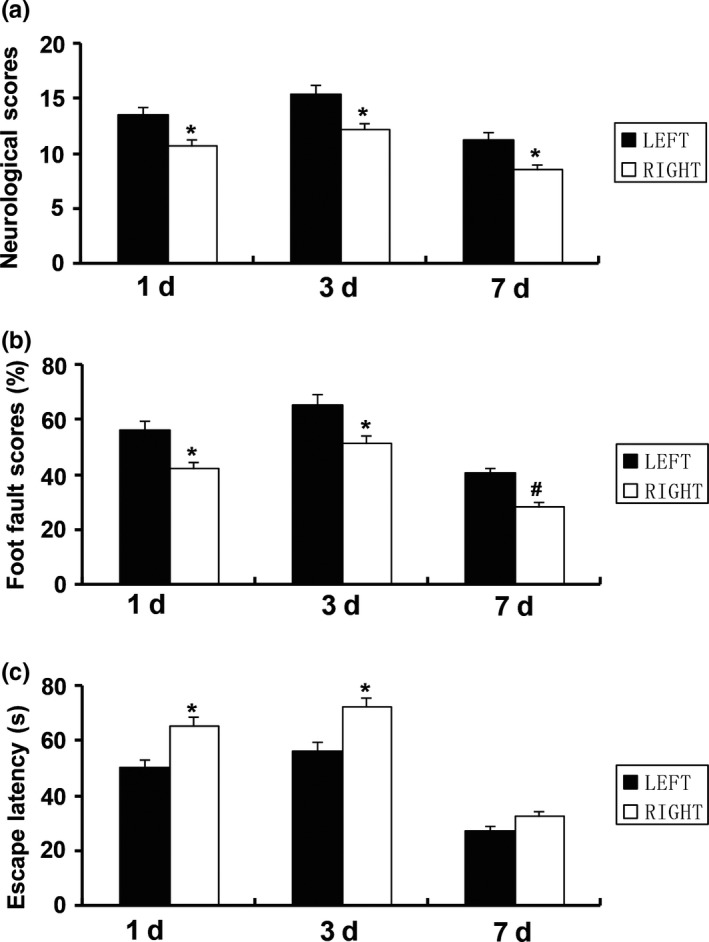
Comparison of neurological deficits between the left and right MCAO groups at 1d, 3d, and 7d after MCAO. (a) mNSS. (b) The foot fault scores in the tapered beam‐walking test. (c) Escape latency in the Morris water maze test. Data are presented as the means ± *SD* (*n *= 6). *p < 0.05, ^#^ *p *< 0.01

The mNSS was significantly higher in the left MCAO group than that in the right MCAO group at 1d, 3d, and 7d after MCAO (13.5 ± 2.16 vs. 10.77 ± 1.81, 15.48 ± 2.73 vs. 12.18 ± 2.09, 11.33 ± 1.64 vs. 8.58 ± 1.42, Figure 1a). Similarly, there was significant statistical difference in the foot fault scores in the tapered beam‐walking test between the two groups. The foot fault scores were higher in the left MCAO group than those in the right MCAO group (56.59 ± 10.03 vs. 42.37 ± 8.2, 65.71 ± 9.78 vs. 51.45 ± 7.28, 40.57 ± 5.59 vs. 28.72 ± 4.75, Figure 1b).

In the water maze test, the left MCAO group rats demonstrated better spatial learning ability than the right MCAO group at 1d and 3d after MCAO (50.44 ± 10.16 vs. 65.27 ± 11.35, 56.55 ± 10.38 vs. 72.3 ± 12.26, Figure 1c). The rats showed similar escape latency, and no differences were achieved between the two groups at 7d after MCAO (27.58 ± 7.51 vs. 32.93 ± 8.24, Figure 1c). These results demonstrated that lesions in the left hemisphere produced more severe sensorimotor impairments, while more severe cognitive impairments were produced by the right hemispherical lesions.

### 3.2 Infarction volume

**Figure 2:**
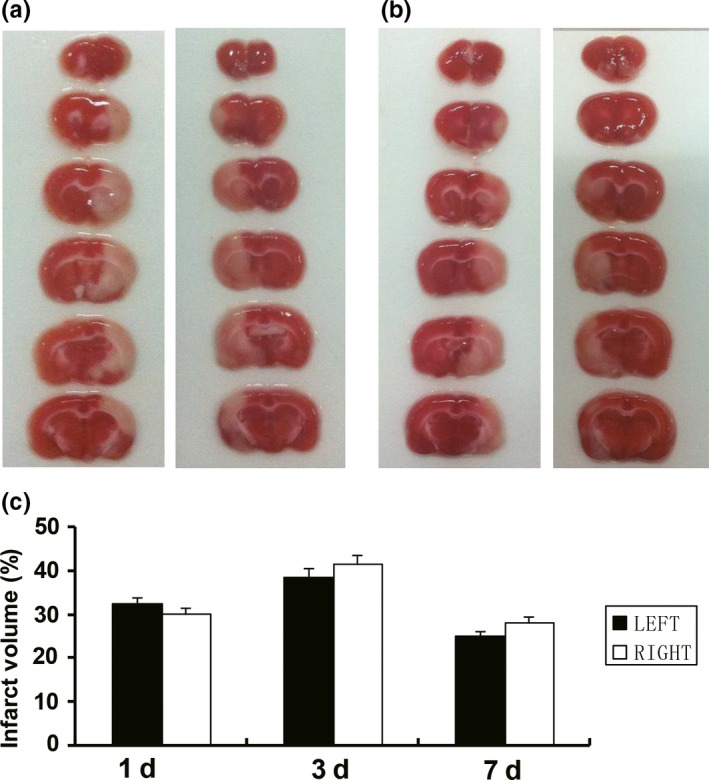
Comparison of infarction volume between the left and right MCAO groups at 1d, 3d, and 7d after MCAO. (a) Representative sets of TTC‐stained brain slices at 3d after the left and right MCAO, respectively. (b) Representative sets of TTC‐stained brain slices at 7d after the left and right MCAO, respectively. (c) The total brain infarct volume was the largest in 3d post‐MCAO rats, and smallest in 7d post‐MCAO rats. There was no significant difference in the infarct volume between the two groups. Data are presented as the mean ± *SD* (*n* = 6)

The cerebral infarction areas were restricted to the frontal, parietal, temporal cortex, and outside parts of striatum supplied by ipsilateral MCA. The total brain infarct volume was the largest in 3d post‐MCAO rats. The smallest infarct volume was found in 7d post‐MCAO rats (Figure 2a,b). However, there was no significant difference in the corrected percentage of infarct volume between the two groups at 1d, 3d, and 7d after MCAO (32.43 ± 3.88 vs. 30.16 ± 3.24, 38.7 ± 3.69 vs. 41.52 ± 4.17, 25.06 ± 2.83 vs. 28.08 ± 2.74, Figure 2c).

## 4 DISCUSSION

In this study, we report the infarct volume and behavioral function following left or right MCAO. A significant increase in mNSS and foot fault score were found in the left MCAO rats, as compared to the MCAO ones. On the other hand, the right MCAO rats showed more higher escape latency. But the infarct volume is not affected significantly by the left or right MCAO model. These data suggest that the group differences observed in the present study were not caused by infarct volume, but rather by structural and functional asymmetry between the two hemispheres.

Middle cerebral artery occlusion models have widely involved rats for stroke‐related research. However, researches about MCAO mostly pay attention to different procedures, such as filament size and tip diameter, the loci of occlusion, thread insert length, reperfusion periods, and surgical techniques. The view that laterality is unique to the human cortex has been supplanted by overwhelming evidence of left to right asymmetry in anatomy and neural processing across vertebrate (Halpern et al., 2005). Research animal models have refocused attention on the advantage of brain laterality. Therefore, asymmetry in the brain should be considered for MCAO model engineering.

In this study, TTC stain demonstrated that the cerebral infarction areas were restricted to the frontal, parietal, temporal cortex, and outside parts of striatum supplied by ipsilateral MCA. So the MCAO models exhibited a comprehensive neurological deficit, including motor impairment, sensory impairment, balance disorder, reflex absence, and abnormal movements. The left hemisphere could play a more important role in the body movement in the right‐handed rats (Robinson, 1979). As expected, our data indicate that a higher increased mNSS in the left MCAO rats, as compared to the right. The foot fault scores in the tapered beam‐walking test were higher in the left MCAO group than those in the right, which further confirmed the above result.

In humans, some hippocampal‐dependent memory functions may be lateralized, the right hippocampus being predominantly involved in spatial navigation. In rodents, the possible lateralization remains unclear. There may be a leftward dominant of hippocampal functions in engram formation or information transfer, and a rightward dominant in spatial memory storage/retrieval processes (Klur et al., 2009). Shinohara et al. (2012) analyzed the performance of “split‐brain” mice in the Barnes maze, the results suggested that the usage of the right hippocampus improves the accuracy of spatial memory. This is consistent with our observation that the right MCAO group rats acquired a longer escape latency to locate the platform in comparison with the left ones at 1d and 3d after MCAO. While the rats showed similar escape latency, and no differences were achieved between the two groups at 7d after MCAO. This is probably because the right MCAO group rats have less motor function damage and therefore get more functional exercise. Some studies found that exercise had been shown to reduce neurological deficits and to ameliorate brain injury in a murine MCAO model (Endres et al., 2003). Furthermore, exercise augmented the recruitment of EPCs into the ischemic region and improved recovery (Gertz et al., 2006).

Gao and Zhang (2008) have previously reported the infarct volume in the dominant hemisphere was larger than that in the non‐dominant hemisphere in adult rats. Interestingly, there was no significant difference in the infarct volume between the two groups in our study at 1d, 3d, and 7d after MCAO, which was consistent with Robinson's researches (Robinson, 1979; Robinson & Coyle, 1980). The comparability of the two hemispheric infarcts maybe ascribed to the symmetric distribution of cerebral arteries in Sprague Dawley rats. Xia et al. (2002) found that the blood flow distributed into both cerebral hemispheres equally in the newborn rats. Therefore, the location of the lesion by MCAO was comparable for the two hemispheric infarcts. The result suggests that infarct volume is not affected significantly by the left or right MCAO model. We also found that the total brain infarct volume was the largest in 3d post‐MCAO rats, the smallest in 7d rats. An early pathogenic event in ischemic brain injury is cytotoxic swelling in which water from the vasculature enters the brain across the BBB (Manley et al., 2000). With the reduction of cerebral edema, the infarction size was shown to be reduced at 7d after MCAO. Exercise also plays a beneficial role (Endres et al., 2003).

Although left‐right asymmetry is a feature of higher‐order brain, little is known about how asymmetry in the brain affect animal behavior. The L‐R asymmetry of hippocampal circuitry is critical for the acquisition of reference memory and the retention of working memory (Goto et al., 2010). The chemical asymmetry including dopamine and norepinephrine also influenced functional asymmetry in adult rat brain (Robinson, 1979).

The limitations of the present study include the statistical limitations of this study due to the small sample size and the insufficiency of mNSS to evaluate sensorimotor performance comprehensively. To confirm the conclusion, tests of sensorimotor performance (e.g., chimney, accelerating rotarod, pole, corner, adhesive removal, or staircase tests) need to be conducted in the future. In addition, due to its inadequate anesthetic properties, marked respiratory depression and extremely irritation of the peritoneum, chloral hydrate is no longer recommended as an anesthetic (Baxter, Murphy, Taylor, & Wolfensohn, 2009). The asymmetry of biochemical response to cerebral infarction can be attributed to differences in the lesion size produced in either hemisphere. Future studies should be directed toward these goals.

In conclusion, our data demonstrate a significant functional asymmetry between the two hemispheres by MCAO model. It is structural and functional asymmetry between the two hemispheres other than infarct volume that affects the outcomes of rat MCAO**.**

## CONFLICT OF INTEREST

None declared.
